# Establishment of a risk prediction model for multidrug-resistant bacteria in deceased organ donors: a retrospective cohort study in China

**DOI:** 10.3389/fcimb.2023.1181630

**Published:** 2023-05-25

**Authors:** Guojie Shen, Li Zhang, Weina Fan, Haifeng Lv, Feifei Wang, Qingqing Ye, Miaozuo Lin, Xia Yu, Hongliu Cai, Xiaoliang Wu

**Affiliations:** ^1^ Department of Intensive Care Unit, The First Affiliated Hospital of Zhejiang University School of Medicine, Hangzhou, China; ^2^ Department of Respiratory, Affiliated Xiaoshan Hospital, Hangzhou Normal University, Hangzhou, China; ^3^ Respiratory Care Department, The First Affiliated Hospital of Zhejiang University School of Medicine, Hangzhou, China; ^4^ Collaborative Innovation Center for Diagnosis and Treatment of Infectious Diseases, The First Affiliated Hospital of Zhejiang University School of Medicine, Hangzhou, China

**Keywords:** multidrug-resistant bacteria, transplantation, organ donors, prediction model, nomogram

## Abstract

**Background:**

Multidrug resistance in bacteria is a serious problem in organ transplantations. This study aimed to identify risk factors and establish a predictive model for screening deceased organ donors for multidrug-resistant (MDR) bacteria.

**Methods:**

A retrospective cohort study was conducted at the First Affiliated Hospital of Zhejiang University School of Medicine from July 1, 2019 to December 31, 2022. The univariate and multivariate logistic regression analysis was used to determine independent risk factors associated with MDR bacteria in organ donors. A nomogram was established based on these risk factors. A calibration plot, receiver operating characteristic (ROC) curve, and decision curve analysis (DCA) were used to estimated the model.

**Results:**

In 164 organ donors, the incidence of MDR bacteria in culture was 29.9%. The duration of antibiotic use ≥3 days (odds ratio [OR] 3.78, 95% confidence interval [CI] 1.62–8.81, p=0.002), length of intensive care unit (ICU) stay per day(OR 1.06, 95% CI 1.02–1.11, p=0.005) and neurosurgery (OR 3.31, 95% CI 1.44–7.58, p=0.005) were significant independent predictive factors for MDR bacteria. The nomogram constructed using these three predictors displayed good predictive ability, with an area under the ROC curve value of 0.79. The calibration curve showed a high consistency between the probabilities and observed values. DCA also revealed the potential clinical usefulness of this nomogram.

**Conclusions:**

The duration of antibiotic use ≥3 days, length of ICU stay and neurosurgery are independent risk factors for MDR bacteria in organ donors. The nomogram can be used to monitor MDR bacteria acquisition risk in organ donors.

## Introduction

Organ transplantation is currently considered the best therapeutic option for patients with end-stage organ failure (Pilmis et al., 2023). However, owing to the growing shortage of organs, there is a stark discrepancy between the number of organs available for transplantation and the number of recipients on the waiting list (Lewis et al., 2021). As many patients die each year while waiting to donate organs, nearly 20% of donors are used for organ transplantation to increase the availability of organs for transplantation, even if they are affected by infectious diseases (Len et al., 2008; Nanni Costa et al., 2008).

With the rapid advancement of organ transplantation, donor-derived infections (DDI) have posed a major challenge to the transplant community while saving the lives of many patients with organ failure. A DDI is defined as any infection in the donor that is transmitted to one or more recipients (Sifri and Ison, 2012; Wolfe et al., 2019). It is an important cause of morbidity in solid organ transplant recipients, including vascular anastomotic dehiscence, infection, and death (Nelson et al., 1984; Anesi et al., 2020). Multidrug-resistant (MDR) bacterial infection is a major public health problem of global magnitude and alarming scale and is also a tougher problem in DDI (Rossolini and Mantengoli, 2008; Lewis and Sifri, 2016). When MDR bacterial DDI occur, they can have serious and devastating results, with mortality rates as high as 33-41% (Ison et al., 2013; Lewis and Sifri, 2016).

There is still much controversy as to whether organs from potential donors colonized or infected with MDR organisms should be accepted (Wolfe et al., 2019). The goal of organ transplantation is to minimize the incidence of DDI while maximizing opportunities for transplantation (White et al., 2019). However, screening potential donors for MDR bacterial infections is not always straightforward and generally requires risk stratification, which relies on the potential organ donor’s social and medical disease history, and needs to be confirmed by culture and drug sensitivity results (Nanni Costa et al., 2008; Fishman and Grossi, 2014). The growth and culture of microorganisms take a long time, leading to an underestimation of the incidence of infection and delay in treatment (Fishman et al., 2012). Therefore, it is critical to identify donors with a higher risk of MDR bacterial colonization or infection before transplantation to facilitate targeted monitoring and treatment of recipients. There are few reliable systems for early assessment of MDR bacterial risk in deceased organ donors to date.

This study aimed to identify the risk factors and establish a predictive model suitable for doctors to screen organ donors with MDR bacteria early and thus select appropriate antimicrobial drugs for recipients to reduce the impact of these bacteria.

## Materials and methods

### Study design

A retrospective cohort study was performed to investigate adult deceased organ donors (≥18 years of age) who donated at least one solid organ at the First Affiliated Hospital of Zhejiang University School of Medicine from July 1, 2019 to December 31, 2022. Eligible donors were identified by the organ procurement organization (OPO). Specimens were cultured during the donor’s terminal hospitalization using blood, urine, perfusate (OPO cultures), rectal swabs, and respiratory secretions. *In vitro* antimicrobial susceptibility testing was performed using the Kirby Bauer paper diffusion method, and the minimum inhibitory concentration (MIC) was determined using the agar dilution method (Jiang et al., 2022). Interpretation of the antimicrobial susceptibility test results followed the recommendations of the Clinical and Laboratory Standards Institute (Humphries et al., 2021). This study was approved by the institutional ethics review board of the First Affiliated Hospital of Zhejiang University.

### Data collection

The preoperative clinicopathological characteristics of all the participants were extracted from the hospital’s medical records. The following items were investigated as possible risk factors: age, gender, hypertension, diabetes mellitus, chronic kidney disease, length of stay in intensive care unit (ICU), death mechanism, procedures during terminal hospitalization, use of proton pump inhibitors (PPIs), enteral feeding, Acute Physiology and Chronic Health Evaluation II (APACHE II) score, dialysis, duration of antibiotic use per donor, and used more than one antibiotic. The events and periods considered in the analysis were recorded before collecting positive biological samples.

### Outcome

We included the following bacteria in our analysis: carbapenem-resistant *Enterobacteriaceae* (CRE), methicillin-resistant *Staphylococcus aureus* (MRSA), extended-spectrum β-lactamase producing *Enterobacteriaceae* (ESBL-PE). All other bacteria (such as *Pseudomonas* species, *Acinetobacter* species, *Enterococcus faecium)* were considered MDR bacteria if they showed resistant to at least one agent in three or more antimicrobial categories (Magiorakos et al., 2012; Jiang et al., 2020). The primary outcome of this study was the culture of MDR bacteria during terminal hospitalization or at the time of organ transplantation (OPO culture). Bacterial cultures from any anatomical site or organ procured were also considered. The distinction between infection and colonization by MDR bacteria has not yet been determined. If MDR bacteria were isolated from the same patient on multiple occasions, only the first episode was considered.

### Statistical analyses and predictive model construction

Statistical analyses were performed using SPSS (version 22.0; IBM SPSS Statistics, IBM Corporation, Armonk, NY, United States) and R software (version 4.2.0; R Foundation for Statistical Computing, Vienna, Austria). Continuous variables were presented as mean ± standard deviation or median and interquartile range (INQ), and Student’s t-test or Wilcoxon test was used for comparisons between groups. Categorical variables are expressed as counts and percentages, and the chi-square test or Fisher’s exact test was used for comparison between groups. Statistical significance was set at a two-tailed p-value of <0.05.

Univariate and multivariate logistic regression analyses were performed to identify the risk factors. Variables with a p-value of <0.1 from univariate analysis was included in a multivariate logistic regression. Effect measures were calculated using odds ratios (OR) and respective 95% confidence intervals (CI). All the selected features were statistically significant and were applied to construct the nomogram prediction model. The “rms” package used to develop the nomogram diagram also uses the R language.

Furthermore, the nomogram performance was assessed using discrimination and calibration. The “pROC” package was used for the ROC curve operation. The area under the curve (AUC) was used to determine the quality of the nomogram. The “rms” package was used to draw and calculate the calibration curves, which were performed by a visual calibration plot comparing the predicted and actual probability of MDR bacteria. The “nricens” package was used for the decision curve analysis (DCA), which is used to determine the clinical practicability of a nomogram based on the net benefit under different threshold probabilities in the deceased organ donors.

## Results

### Characteristics of the study cohort

**Figure 1:**
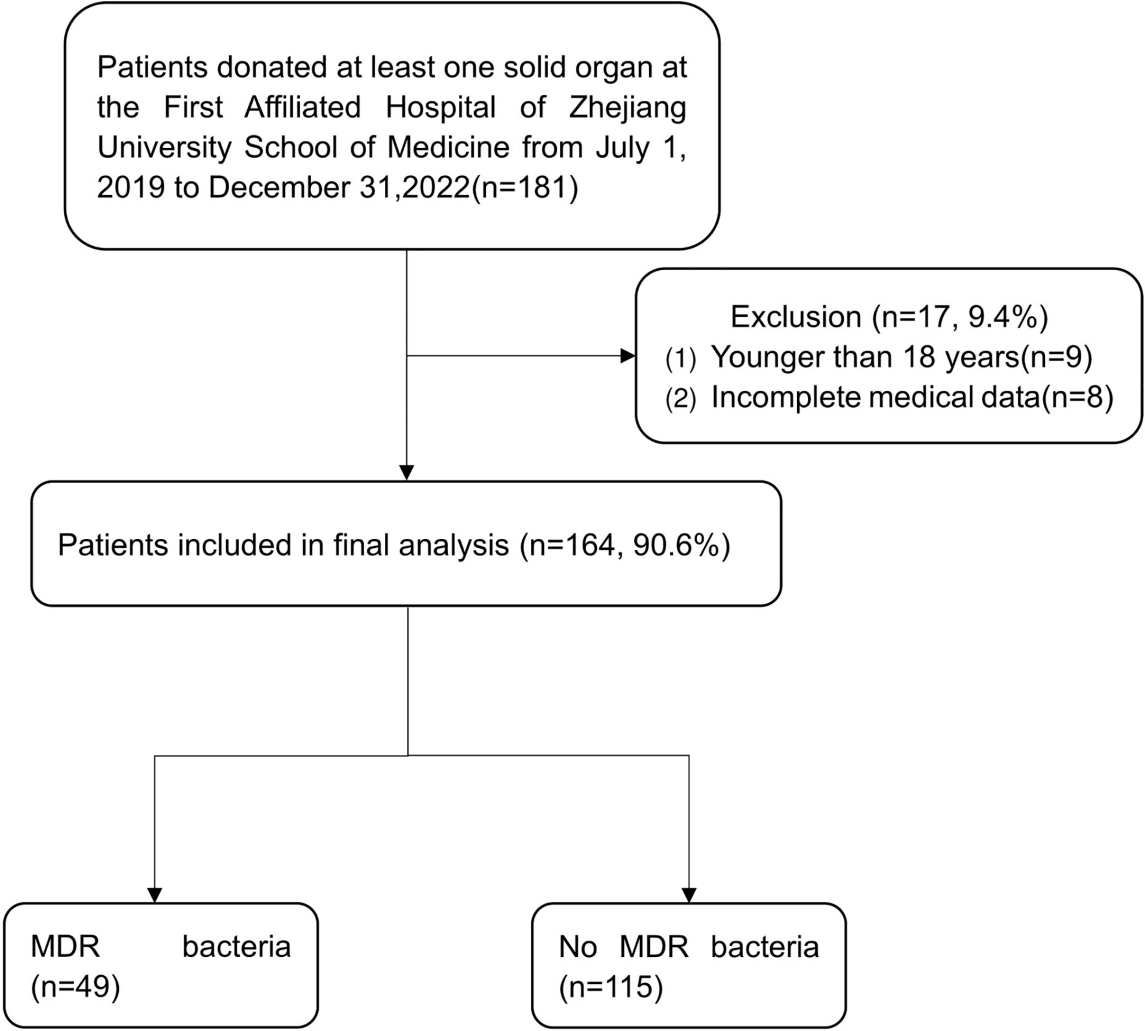
Flow chart of study design.

In our study, 181 cases with at least one solid organ donated were admitted to the First Affiliated Hospital of Zhejiang University School of Medicine from July 1, 2019 to December 31, 2022. Nine patients with incomplete medical data and eight patients younger than 18 years of age were excluded. A flow diagram of the study design is shown in **Figure 1**. 138 (84.1%) were male and 26 (15.9%) were female, with a median age of 46 years (INQ 33-54). Common comorbidities included hypertension (n = 43, 26.2%), diabetes mellitus (n = 10, 6.1%), and chronic kidney disease (n = 4, 2.4%). The median length of stay in ICU was 8 days (INQ 5-13). The main death mechanisms were intracranial hemorrhage (71, 43.3%), traumatic brain injury (70, 42.7%), and cerebral infarction (8, 4.9%). Others (15, 9.1%) included asphyxiation (5, 3.0%), drowning (3, 1.8%), drug intoxication (3, 1.8%), pulmonary embolism (2, 1.2%), hypoglycemia (1, 0.6%), and myocardial infarction (1, 0.6%). 49 (29.9%) cases isolated MDR bacteria during terminal donor hospitalization or at the time of organ transplantation. The additional basic patient characteristics are presented in **Table 1**.

**Table 1:** Characteristics of deceased organ donors stratified by MDR bacteria status.

Characteristic	Total	No MDR bacteria	MDR bacteria	p
Gender				
Male	138(84.1%)	95(82.6%)	43(87.8%)	0.409
Female	26(15.9%)	20(17.4%)	6(12.2%)	
Age, years	46(33-54)	48(37-55)	43(29-52)	0.061
Length of stay in ICU, days	8(5-13)	6(4-10)	12(8-22)	<0.001^*^
Comorbidities				
Hypertension	43(26.2%)	32(27.8%)	11(22.4%)	0.474
Diabetes mellitus	10(6.1%)	8(7.0%)	2(4.1%)	0.725
Chronic kidney disease	4(2.4%)	3(2.6%)	1(2.0%)	1
Death mechanism				
Traumatic brain injury	70(42.7%)	45(39.1%)	25(51.0%)	0.159
Intracranial hemorrhage	71(43.3%)	53(46.1%)	18(36.7%)	0.269
Cerebral infarction	8(4.9%)	7(6.1%)	1(2.0%)	0.438
Other	15(9.1%)	10(8.7%)	5(10.2%)	0.759
Procedures during terminal hospitalization				
Neurosurgery	62(37.8%)	38(33.0%)	24(49.0%)	0.054
Tracheostomy	7(4.3%)	4(3.5%)	3(6.1%)	0.427
Open abdomen	6(3.7%)	6(5.2%)	0	0.18
Use of PPIs	154(93.9%)	109(94.8%)	45(91.8%)	0.471
Enteral feeding	64(39.0%)	41(35.7%)	23(46.9%)	0.175
APACHII score	26.9 ± 5.8	27.0 ± 5.5	26.6 ± 6.4	0.66
Dialysis	17(10.4%)	11(9.6%)	6(12.2%)	0.606
Previous antibacterial therapy	153(93.3%)	106(92.2%)	47(95.9%)	0.38
Penicillin antibiotics	91(55.5%)	65(56.5%)	26(53.1%)	0.683
Second generation cephalosporins	24(14.6%)	18(15.7%)	6(12.2%)	0.572
Third or fourth generation cephalosporins	37(22.6%)	22(19.1%)	15(30.6%)	0.107
Carbapenems	36(22.0%)	22(19.1%)	14(24.6%)	0.181
Quinolones	7(4.3%)	4(3.5%)	3(6.1%)	0.427
Duration of antibiotic use per donor,days	2.5(2-5)	2(2-4)	5(3-10)	<0.001^*^
≥3	62(37.8%)	30(18.3%)	32(19.5%)	<0.001^*^
<3	102(62.2%)	85(51.8%)	17(10.4%)	
Used more than one antibiotic	58(35.4%)	34(29.6%)	24(49.0%)	0.017^*^

MDR, Multidrug-resistant; ICU, Intensive Care Unit; PPIs, Proton Pump Inhibitors; APACHE II, Acute Physiology and Chronic Health Evaluation II.

^*^represents p <0.05.

**Table 2** lists the classification and percentage of 49 (29.9%) cases that isolated MDR bacteria. Eight MDR bacteria species were cultured in 164 cases. From these cases, gram-negative bacteria were isolated in 42 (25.6%) patients, with a predominance of *Acinetobacter baumannii*, *Klebsiella pneumonia*, and *Pseudomonas aeruginosa*, and gram-positive bacteria from 7 (4.3%) patients were cultured, with *Staphylococcus aureus*, and *Enterococcus faecium* as the main bacteria. The predominant sites of infection in first-episode MDR bacteria are respiratory secretions and blood cultures.

**Table 2:** Overview of MDR bacteria isolated from the 164 cases and the source of each isolate.

Bacteria type	Site of growth	N(%)
GN bacteria		42(25.6%)
*Acinetobacter baumannii*	Donors with *A. baumannii* on culture	18(11.0%)
	Respiratory cultures with *A. baumannii*	16
	Perfusate cultures with *A. baumannii*	2
*Klebsiella pneumoniae*	Donors with *K. pneumoniae* on culture	10(6.1%)
	Blood cultures with *K. pneumoniae*	5
	Respiratory cultures with *K. pneumoniae*	2
	Rectal swab cultures with *K. pneumoniae*	2
	Urine cultures with *K. pneumoniae*	1
*Pseudomonas aeruginosa*	Donors with *P. aeruginosa* on culture	6(3.7%)
	Respiratory cultures with *P. aeruginosa*	5
	Rectal swab cultures with *P. aeruginosa*	1
*Escherichia coli*	Donors with *E. coli* on culture	5(3.0%)
	Respiratory cultures with *E. coli*	2
	Perfusate cultures with *E. coli*	2
	Rectal swab cultures with *E. coli*	1
*Serratia marcescens*	Donors with *S. marcescens* on culture	2(1.2%)
	Respiratory cultures with *S. marcescens*	1
	Urine cultures with *S. marcescens*	1
*Enterobacter cloacae*	Donors with *E. cloacae* on culture	1(0.6%)
	Respiratory cultures with *E. cloacae*	1
GP bacteria		7(4.2%)
*Staphylococcus aureus*	Donors with *S. aureus* on culture	5(3.0%)
	Respiratory cultures with *S. aureus*	4
	Blood cultures with *S. aureus*	1
*Enterococcus faecium*	Donors with *E. faecium* on culture	2(1.2%)
	Perfusate cultures with *E. faecium*	1
	Blood cultures with *E. faecium*	1

GN, Gram-negative; GP, Gram-positive.

### Predictive model construction

**Figure 2:**
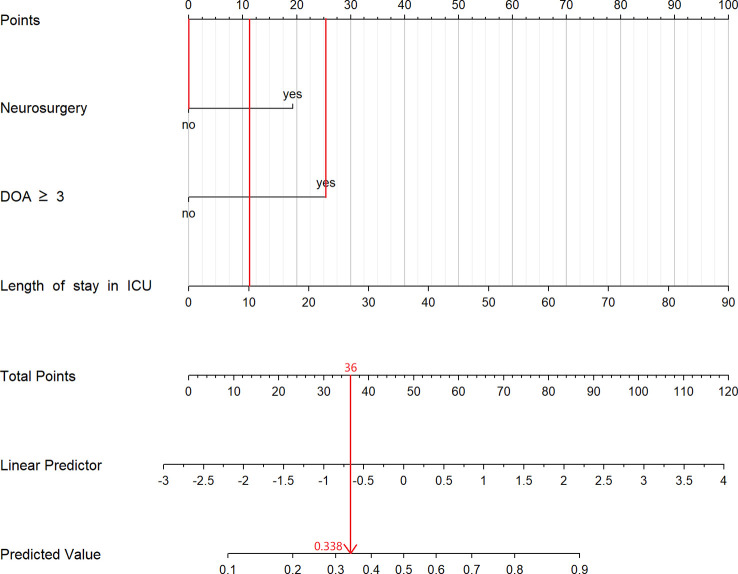
The constructed nomogram for predicting risk of MDR bacteria in organ donors. This patient stayed in ICU for 10 days, used antibiotic more than 3 days and had not experienced neurosurgery. According to the nomogram, we can calculate that the total point for this patient is 36 and its corresponding risk is 33.8%. DOA, duration of antibiotic; ICU, Intensive Care Unit.

Univariate and multivariate logistic analyses were used to identify the potential risk factors. Multivariable analyses demonstrated that the occurrence of MDR bacteria was significantly correlated with The duration of antibiotic use ≥3 days (odds ratio [OR] 3.78, 95% confidence interval [CI] 1.62–8.81, p=0.002), length of ICU stay,days(OR 1.06, 95% CI 1.02–1.11, p=0.005) and neurosurgery (OR 3.31, 95% CI 1.44–7.58, p=0.005). The detailed results of univariate and multivariate analyses are presented in **Table 3**. Based on regression analysis, a nomogram was constructed to quantitatively predict the risk probability of MDR bacteria in patients with deceased organ donors. Each value of these variables is assigned a score on the point-scale axis. The total score can be easily calculated by summing the individual scores. **Figure 2** gave an example to show how the nomogram could be used as a prognostic stratification tool to estimate the probability of MDR bacteria in organ donors.

**Table 3:** Univariate and multivariate logistic regression analysis of the predictors for MDR bacteria in deceased organ donors.

	Univariate analysis			Multivariate analysis		
	OR	95%CI	p	OR	95%CI	p
Gender (female VS male)	0.663	0.249-1.767	0.411			
Age, years	0.975	0.951-1.001	0.055	0.979	0.950-1.008	0.16
Length of stay in ICU, days	1.085	1.042-1.131	<0.001^*^	1.063	1.017-1.112	0.005^*^
Hypertension	0.751	0.342-1.647	0.474			
Diabetes mellitus	0.569	0.116-2.783	0.486			
Chronic kidney disease	0.778	0.079-7.667	0.830			
Traumatic brain injury	1.620	0.826-3.179	0.160			
Intracranial hemorrhage	0.679	0.342-1.350	0.270			
Cerebral infarction	0.321	0.038-2.685	0.295			
Neurosurgery	1.945	0.984-3.846	0.056	3.308	1.443-7.583	0.005^*^
Tracheostomy	1.810	0.390-8.407	0.449			
Open abdomen	0.000	0.000	0.999			
Use of PPIs	0.619	0.167-2.300	0.474			
Enteral feeding	1.597	0.810-3.147	0.177			
APACHII score	0.987	0.931-1.046	0.658			
Dialysis	1.319	0.459-3.794	0.607			
Previous antibacterial therapy	1.995	0.415-9.593	0.389			
Penicillin antibiotics	0.870	0.444-1.702	0.683			
Second generation cephalosporins	0.752	0.279-2.026	0.573			
Third or fourth generation cephalosporins	1.865	0.868-4.007	0.110			
Carbapenems	1.691	0.779-3.669	0.184			
Quinolones	1.810	0.390-8.407	0.449			
Duration of antibiotic use ≥3 days	5.333	2.594-10.963	<0.001^*^	3.780	1.623-8.805	0.002^*^
Used more than one antibiotic	2.287	1.149-4.553	0.019^*^	1.038	0.449-2.400	0.931

MDR, Multidrug-resistant; ICU, Intensive Care Unit; PPIs, Proton Pump Inhibitors; APACHE II, Acute Physiology and Chronic Health Evaluation II.

^*^represents p <0.05.

### Predictive model validation

**Figure 3:**
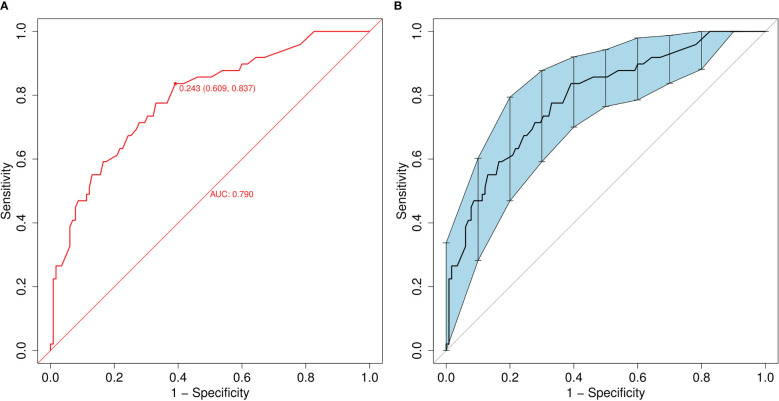
**(A)** Receiver operating characteristic (ROC) curve of the nomogram model for predicting the risk of MDR bacteria. The y-axis represents the true positive rate of the risk prediction, and the x-axis represents the false positive rate of the risk prediction. **(B)** ROC of the predictive MDR bacteria risk nomogram using 200 bootstrap re-samples.

**Figure 4:**
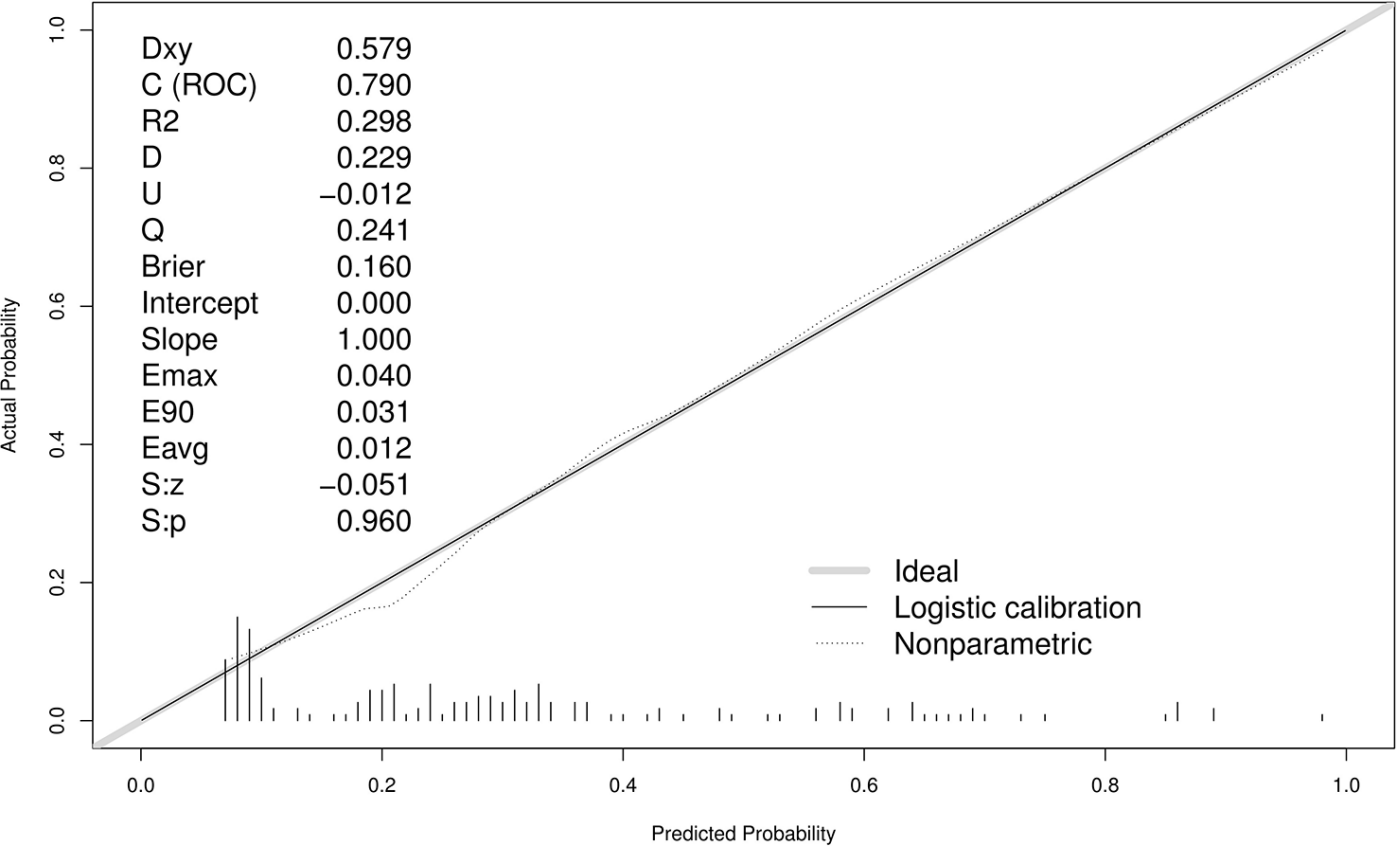
Calibration curves of the predictive MDR bacteria risk nomogram using 500 bootstrap re-samples. The y-axis represents actual diagnosed cases of MDR bacteria, and the x-axis represents the predicted risk of MDR bacteria. The diagonal dotted line represents a perfect prediction by an ideal model, while the solid line represents the performance of the data.

**Figure 5:**
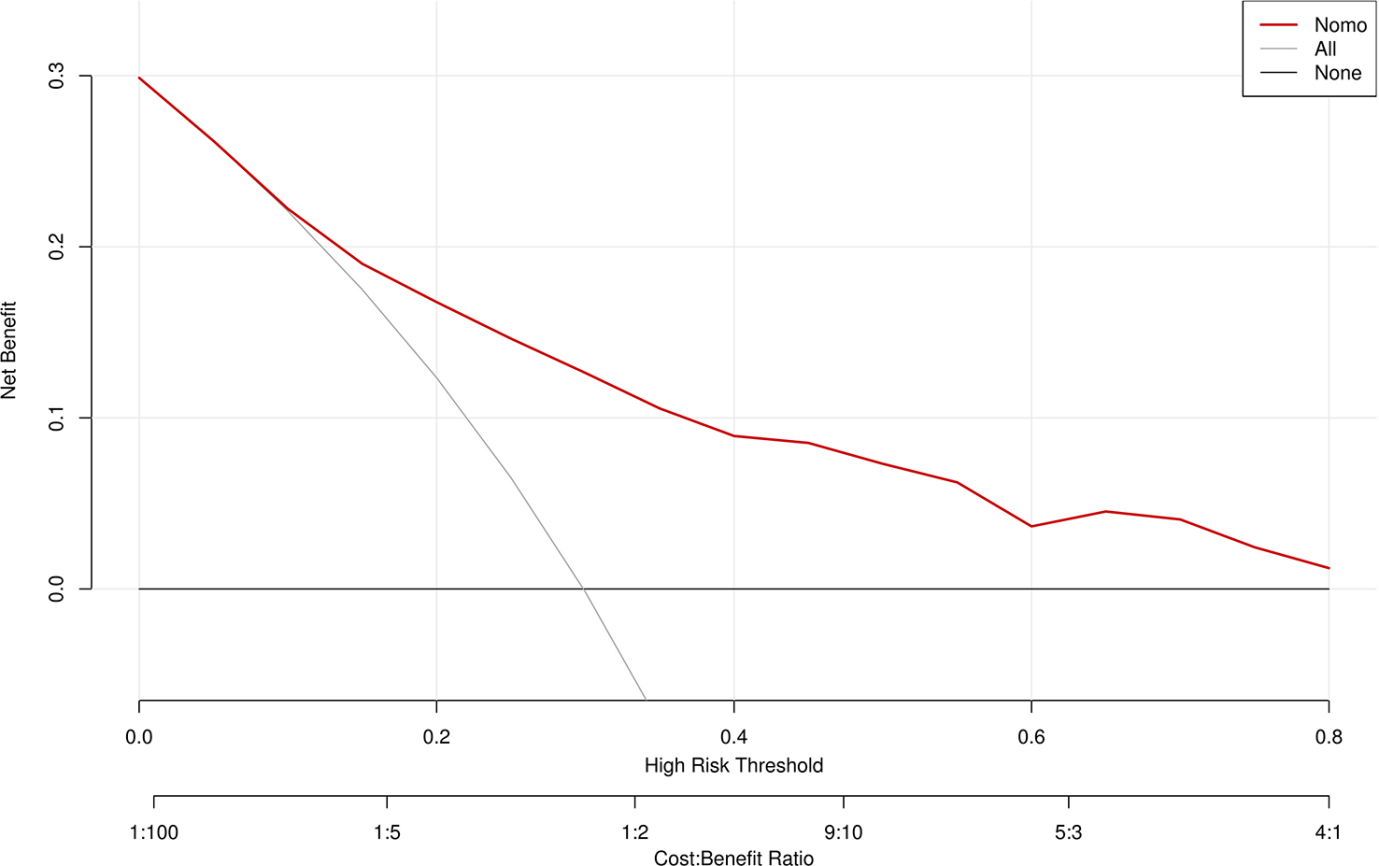
Decision curve analysis for the MDR bacteria risk nomogram. The y-axis measures the net benefit. The thick solid line represents the assumption that all patients have no MDR bacteria, the thin solid line represents the assumption that all patients have MDR bacteria, and the dotted line represents the risk nomogram.

A ROC curve was used to evaluate the discriminatory capacity of the predictive model. For the predictive model, the pooled AUC of the nomogram was 0.79 (95%CI: 0.71-0.87), indicating a moderately good performance (**Figures 3A, B**). A calibration plot was developed to determine the predictive capacity of the nomograms using 500 bootstrap re-samples. **Figure 4** illustrates that the predictive model and validation set closely adhere to the reference line. DCA demonstrated that this model could increase the net benefits and show a wide range of threshold probabilities (**Figure 5**).

## Discussion

This retrospective cohort study found that the duration of antibiotic use ≥3 days, length of ICU stay and neurosurgery were risk factors for MDR bacterial infection or colonization in deceased organ donors. Based on previous studies, we collected as many predictors as possible associated with MDR bacteria. Deceased organ donors were almost always admitted to the ICU, experienced mechanical ventilation, indwelling central venous catheters, nasogastric tubes, urinary catheters; other possible risk factors, such as enteral nutrition, use of PPIs and antibiotics, surgery, age, gender, etc., were considered (Augustin et al., 2010; Vasudevan et al., 2014; Yamamoto et al., 2017). In this process, various confounding factors must be considered as variables. We determined that multivariate logistic regression analysis provides a good solution to this problem. A nomogram incorporating these three prognostic factors to predict the incidence of MDR bacteria was established and evaluated using ROC curve, calibration, and DCA, providing good predictive accuracy and discriminative ability.

Deceased organ donors may be accompanied by hemodynamic instability and a higher risk of infection, thus extending the duration of antibiotic administration. This has led to the overuse of antibiotics, which may be critical to the development of bacterial resistance (Christaki et al., 2020). In univariate and multivariate logistic analyses, we found a consistent association between the duration of antibiotic use ≥3 days and the emergence of MDR bacteria. This is consistent with previous findings that reducing the duration of antibiotic use leads to a lower incidence of MDR bacteria (Tacconelli et al., 2014; van Langeveld et al., 2017). A randomized trail conducted by Singh et al. revealed no difference in mortality or ICU length of stay for 3 days of empirical antibiotic therapy compared to those who received longer antibiotic therapy (Singh et al., 2000).Shorter course of antibiotic therapy leads to fewer subsequent superinfections attributed to antibiotic-resistant pathogens (Chastre et al., 2006).Thus, continuous monitoring of the duration of antibiotic use may provide a key warning indicator for predicting the increased incidence of drug-resistant bacteria.

In addition, our study found that potential donors had a terminal hospital stay of 8 days (INQ 5–13), which was also longer than that in other studies (Ye et al., 2017; Anesi et al., 2020). Previous studies have shown an association between a prolonged ICU stay and the occurrence of MDR bacteria (Chavada et al., 2018). Our study obtained similar result from the univariate and multivariate analysis. Therefore, minimizing the inappropriate use of antibiotics and shortening the hospitalization time of potential organ donors are important to reduce the incidence of MDR bacteria.

In our study, recent neurosurgery was also a risk factor for the development of MDR bacteria, and a few studies have included data on antimicrobial resistance in the neurocritical care population (Abulhasan et al., 2020). Surgery, an invasive procedure, may lead to an increased risk of bacterial infection; other studies have reported similar results (Mariappan et al., 2017; Lin et al., 2022). Agarwal et al. studied 330 neurosurgical patients with healthcare-associated infections and reported an overall incidence of infection of 6.67%; however, all isolates (100%) were MDR (Agarwal et al., 2017). This may be related to changes in sensory organ function in potential donors and the long-term use of medical devices.

The advantage of our study is that we identified the risk factors and established the first risk prediction model for the onset of MDR bacteria in deceased organ donors in China. The nomogram we constructed enabled healthcare professionals to easily calculate the MDR bacterial acquisition risk for potential donors, allowing them to assess changes in their risk frequently. Therefore, it will facilitate the early detection of patients with high-risk MDR bacterial colonization or infection so recipients can develop individualized strategies in the perioperative period to contain the emergence and spread of MDR strains and reduce their impact.

Our study has several limitations. First, This was a retrospective study and the sample size of this study was limited; the risk prediction model has not been validated with external data, and its generalization performance is unclear. Second, other risk factors have not been identified. Deceased donors are necessarily admitted to the ICU with indwelling tracheal intubation, urinary catheters, nasogastric tubes, central venous catheters, and mechanical ventilation, making none of these factors available as covariates included in the analysis. Finally, these data were collected from a single center. Our results may not be extrapolated to all regions because MDR bacterial epidemiology depends on local conditions and shows a high degree of variation between countries and regions.

## Conclusion

Our study developed the first predictive tool to identify the risk of MDR bacteria in deceased organ donors using three factors: the duration of antibiotic use ≥3 days, length of ICU stay and neurosurgery. Targeted antibiotic therapy is required for organ donors while avoiding the overuse of antibiotics. The nomogram is easy to apply and can help clinicians select the appropriate empirical antibiotic therapies for recipients.

## Data availability statement

The original contributions presented in the study are included in the article/**Supplementary Material**. Further inquiries can be directed to the corresponding author.

## Ethics statement

The studies involving human participants were reviewed and approved by clinical research ethics committee of the First Affliated Hospital, College of Medicine, Zhejiang University.(No. 2022-705). The patients/participants provided their written informed consent to participate in this study. Written informed consent was obtained from the individual(s) for the publication of any potentially identifiable images or data included in this article.

## Author contributions

XW had full access to all of the data in the study and took responsibility for the integrity of the data and the accuracy of the data analysis. Study design: GS and HC. Data collection: HL, FW, WF, QY and ML. Statistical analysis: GS and XY. Manuscript draft: GS and LZ. Manuscript revised: XW. All authors contributed to the article and approved the submitted version.
